# Pattern and Predictors of Infection Among Patients With Rheumatological Disease on Immunosuppressive Medications: A Retrospective Study in a Tertiary Care Hospital in Bangladesh

**DOI:** 10.7759/cureus.52817

**Published:** 2024-01-23

**Authors:** S.K. Jakaria Been Sayeed, Md Moniruzzaman, A K M Humayon Kabir, Md Uzzwal Mallik, Bikas Chandra Mondal, Shahin Mahmud, Fahim T Rahman, Mehrin Rahman, Md. Mujibur Rahman

**Affiliations:** 1 Medicine and Rheumatology, National Institute of Neurosciences and Hospital, Dhaka, BGD; 2 Medicine, Dhaka Medical College Hospital, Dhaka, BGD; 3 Respiratory Medicine, National Institute of Chest Diseases and Hospital, Dhaka, BGD; 4 Rheumatology, National Institute of Neurosciences and Hospital, Dhaka, BGD; 5 Medicine, Popular Medical College Hospital, Dhaka, BGD

**Keywords:** predictors, pattern, immunomodulators, rheumatological diseases, infection

## Abstract

Background

Immunomodulatory therapy for chronic rheumatic disease carries a risk for infectious complications. In Bangladesh, there is limited information regarding patterns and factors associated with infections among patients receiving immunosuppressive medications.

Objective

The present study aimed to find out patterns and predictors associated with infection among patients who were on different immunosuppressive medications due to chronic rheumatological disease.

Methodology

This was a retrospective study; all confirmed cases of (new and old) different rheumatological diseases on disease-modifying agents attended at the rheumatology clinic of Dhaka Medical College Hospital from January 2019 to December 2021 were enrolled.

Result

Among 489 cases, 90 (18.4%) patients had documented infections. The most common rheumatological diseases were systemic lupus erythematosus (28, 31.1%), ankylosing spondylitis (26, 28.8%), and rheumatoid arthritis (20, 22.2%). COVID-19 (28, 31.1%) was the most commonly occurring infection followed by urinary tract infection (14, 15.6%), fungal infection (12, 13.3%), herpes zoster (10, 11.1%), pulmonary tuberculosis (TB) (eight, 8.8%), latent TB (seven, 7.7%), community-acquired pneumonia (six, 6.6%), and sepsis (three, 3.3%). Infection was most prevalent among patients who received steroids of more than 10 mg per day (17, 18.8%) than those less than 10 mg steroid per day (six, 6.7%), Factors associated with infections were (odds ratio, 95% CI, p-value) underweight (2.3, [1.3-2.7], 0.001), anemia (1.8, [1.1-5.7], 0.01), neutropenia (1.6, [1.1-2.9], <0.002), hypoalbuminemia (3.1, [1.6-4.9], 0.001), hypovitaminosis D (1.9, [1.3-4.5], 0.001), high blood sugar (1.5, [1.1-5.3], 0.02), inadequate counseling of steroid side effect (1.7, [1.1-3.9], 0.03), prednisolone >10mg/day (2.2, [1.19-4.10], 0.001).

Conclusion

COVID-19 pneumonia, urinary tract infections, fungal infection, tuberculosis, herpes zoster, and community-acquired pneumonia were commonly occurring infections among patients receiving different immunosuppressive medications. Factors like poor nutritional status, presence of anemia, leucopenia, hypoalbuminemia, hyperglycemia, and hypovitaminosis D had a significant association with infection. Moreover, inadequate counseling of steroid side effects and history of daily intake of prednisolone (>10mg/day) were also significant factors associated with infection.

## Introduction

Management of chronic inflammatory arthritis has been changed vastly due to the disease-modifying anti-rheumatic agents or biological agents. Their efficacy in respective diseases is well established by many clinical trials [1]. Infections are quite common among this group of patients due to the disease itself or Immunomodulatory medications. The risk of infection is higher among patients who are taking biological agents like Janus-associated kinase (JAK-2) inhibitors, tumor necrosis factor alpha inhibitors, and long-time steroids. However, the infection rate is less frequently observed in the conventional disease-modifying antirheumatic drug (DMARD) [2,3]. Different opportunistic infections like tuberculosis, herpes zoster, local and systemic fungal infections, and even COVID-19 are quite common among patients on biological or immunomodulators [4]. Bangladesh is a lower middle-income country where rheumatic diseases are neglected health problems and on some occasions, it is overlooked. Infection related to synthetic DMARDs, biological agents, and steroids is mostly missed. Till now, studies related to patterns and factors that might increase infection among this group of patients are limited in Bangladesh. The aim of the study was to see the infection patterns and determine the predictors of infection among patients with different rheumatological diseases on immunosuppressant medications.

## Materials and methods

The retrospective study was conducted at the rheumatology clinic of Dhaka Medical College from January 2019 to December 2021 using a data registry software system. All diagnosed case of the different rheumatological diseases on immunosuppressive medications with documented infection was included. The objectives were to evaluate the pattern of infection and find out factors associated with infection in this group. A total of 90 patients who had documented infection were enrolled. Blood tests like complete blood count (CBC), inflammatory marker C-reactive protein (CRP), erythrocyte sedimentation rate (ESR), ferritin, blood and urine culture, urine microscopic examination, reverse transcription polymerase chain reaction (RT-PCR) for COVID-19, Chest X-ray, ultrasonogram of the abdomen, hepatitis B surface antigen (HBsAg), liver enzymes, tuberculin skin test, sputum for acid-fast bacillus (AFB), gram stain and gene expert were considered for as conclusive evidence of infection. For the reactivation of tuberculosis, only tuberculin tests were done because interferon-γ release assay (IGRA) is still not available in many centers of Bangladesh. All data were analyzed by SPSS 23 version (IBM Corp., Armonk, NY, USA). Descriptive statistics were reported as mean and standard deviation for continuous variables, numbers, and percentages for categorical variables. To compare variables with infection, a chi-square test for qualitative value, and for quantitative value student t-test were done (normal distribution). However, for values with skewed deviation Mann-Whitney U test was done. For predictor analysis, logistic regression analysis was done, expressed as an odds ratio with a 95% confidence interval where p-value <0.05 was considered significant. As it was conducted by using data registration software of the Rheumatology Clinic, only ethical permission was taken from the Ethics Review Committee (ERC) of Dhaka Medical College Hospital. Informed consent from the patient was not taken.

## Results

Among 489 cases, 90 (18.4%) patients had documented infections from 2019 to 2021. The mean age was 33 (±9), and 60 (66.7%) were female. The mean BMI was 19.3 (±7.6) kg/m2 and among them, 39 (43.3%) had BMI <18.5 kg/m2. Hypertension (22, 24.4%) and diabetes (12, 13.3%) were the most common comorbidities. The most common rheumatic diseases were systemic lupus erythematosus (28, 31.1%), ankylosing spondylitis (26, 28.8%), and rheumatoid arthritis (20, 22.2%). The most commonly prescribed conventional synthetic DMARD were hydroxychloroquine (32, 35.5%), methotrexate (32, 35.5%), and sulfasalazine (30, 33.3%). Mycophenolate mofetil (14, 15.5%), cyclophosphamide (10, 11.1%), and azathioprine (six, 12.2%) were the most common immunomodulatory drugs. The most commonly used biological agents were tofacitinib (10, 11.1%), and adalimumab (six, 6.7%). However, 40 (44.4%) patients took prednisolone, for a mean duration of 4.3 (±1.3) months. COVID-19 (28, 31.1%) was the most commonly occurring infection followed by urinary tract infection (UTI) (14, 15.6%), fungal infection (12, 13.3%), herpes zoster (10, 11.1%), pulmonary tuberculosis (TB) (eight, 8.8%), latent TB (seven, 7.7%), community-acquired pneumonia (six, 6.6%), and sepsis (three, 3.3%) (Table 1).

**Table 1:** Demographic, clinical characteristics with medications and infection pattern among the patients (N= 90) Post CHIK CIR- Post chikungunya chronic inflammatory rheumatism

Trait	Result
Age (Years, Mean ± SD)	33 (± 9)
Sex (Frequency, %)	
Male	30 (33.3)
Female	60 (66.6)
Smoker (Frequency, %)	9 (10)
BMI (kg/m2, Mean ± SD)	19.3 (± 7.6)
< 18.5	39 (43.3)
18.5- 22.9	25 (27.7)
23-24.9	18 (20)
> 25	8 (8.9)
Comorbidities (Frequency, %)	
Hypertension	22 (24.4)
Diabetes	12 (13.3)
Hypothyroidism	7 (7.7)
Hyperthyroidism	1 (1.1)
Asthma	5 (5.5)
Chronic kidney disease	4 (4.4)
Diagnosis (Frequency, %)	
Lupus nephritis	19 (21.1)
Systemic lupus erythematosus	9 (10)
Rheumatoid arthritis	20 (22.2)
Spondyloarhtropathy	26 (28.9)
Systemic Sclerosis	3 (3.3)
Post CHIK CIR	3 (3.3)
Granulomatosis with polyangitis	2 (2.2)
Takayasu Arteritis	2 (2.2)
Mixed connective tissue disease	2 (2.2)
Polymyositis	1 (1.1)
Sjogren Syndrome	2 (2.2)
Psoriatic Arthritis	2 (2.2)
Overlap Syndrome	2 (2.2)
Medications	
Methotrexate	32 (35.5)
Hydroxychloroquine	32(35.5)
Sulfasalazine	30(33.3)
Prednisolone	40(44.4)
Mycophenolate mofetil	14(15.5)
Cyclophosphamide	10(11.1)
Tofacitinib	10(11.1)
Adalimumab	6(6.7)
Azathioprine	6(6.7)
Rituximab	1(1.1)
Infection	
Covid-19	28(31.1)
Urinary tract infection	14(15.6)
Fungal infection	12(13.3)
Herpes zoster	10(11.1)
Pulmonary Tuberculosis	8(8.8)
Latent Tuberculosis	7(7.7)
Pneumonia	6(6.6)
Dental abscess	1(1.1)
Sepsis	3(3.3)

**Figure 1:**
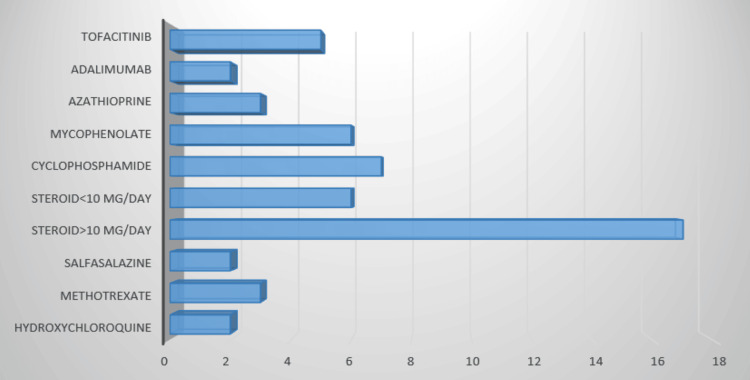
Frequency of infection among patients who received different immunomodulators and biologics (frequency is expressed in number) Steroid means prednisolone.

Infection was most prevalent among patients who received steroids of more than 10 mg per day (17, 18.8%) followed by cyclophosphamide (seven, 7.8%), mycophenolate (six, 6.7%), tofacitinib (five, 5.6%). However, methotrexate (three, 3.3%), sulfasalazine (two, 2.2%), and hydroxychloroquine (two, 2.2%) groups suffered less infection (Figure 1).

Laboratory investigations revealed mean hemoglobin 9.7g/dl (±2.9), median ESR and CRP were 57mm (min 13, max 132) and 42ng/ml (min 9, max 77), ferritin 289ng/ml (±65), vitamin D 25 IU/ml (±11) and hemoglobin A1C (HbA1C) 8.5% (±2.7) (Table 2). 

**Table 2:** Laboratory information among patients with different rheumatological diseases with infection (n=90) ESR- erythrocyte sedimentation rate, CRP- C reactive protein, C3 & C4 - complement, ALT- alanine amino transferase, HbA1C- hemoglobin A1C

Trait	Value
Hemoglobin	9.7 [2.9]
Neutrophil [K/μL, SD]	5.6 [2.7]
Lymphocyte [K/μL, SD]	0.8 [0.4]
Platelet [K/μL, SD]	2.43 [1.24]
CRP (n= 0-10 ng/ml)	57 [min 13, max 132]
ESR ( n= 10-20 mm in 1^st^ hour)	42 [ min 9, max 77]
Proteinuria (n, %)	17 (18.8)
Pus Cell in Urine (n, %)	19 (21.1)
Albumin (n, 3.5-5.5 g/dl)	3.6 (1.3)
HbA1C (< 6%)	8.9 (2.7)
Vitamin D3 (n= 30-50 Iu/ml)	25 ( 11)
Creatinine (n= 0.5-1.2 mg/dl)	0.8 (0.4)
Tuberculin skin Test ( n= 5-15 mm)	13 (7)
Ferritin (n=10-120 ng/ml)	289 (65)
ALT ( n=10-40 IU)	28 (11)
C3 ( N= 0.9-1.8 g/ L)	1.1 (0.4)
C4 (N= 0.2-0.5 g/L)	0.4 (0.07)

Factors associated with infections were (odds ratio, 95% CI, p-value) underweight (2.3, [1.3-2.7], 0.001), anemia (1.8, [1.1-5.7], 0.01), neutropenia (1.6, [1.1-2.9], <0.002), hypoalbuminemia (3.1, [1.6-4.9], 0.001), hypovitaminosis D (1.9, [1.3-4.5], 0.001), high blood sugar 1.5, [1.1-5.3], 0.02), inadequate counseling of steroid side effect (1.7, [1.1-3.9], 0.03), and prednisolone >10 mg/day (2.2, [1.19-4.10], 0.001) (Table 3).

**Table 3:** Predictors of infection among patients on different DMARD or steroids Logistic regression was done to identify the predictors. Predictors were expressed with odds ratio, a P value <0.05 is considered to be significant. DMARD- Disease-modifying antirheumatic drugs

Trait	Odds Ratio [95% CI]	P value
Age	0.3 [0.12-1.6]	0.43
Sex	0.5 [0.21-1.80]	0.67
Smoking	0.87 [0.69-1.86]	0.28
Underweight	2.3 [1.3-2.7]	0.001
Anaemia	1.8 [1.1-5.7]	0.01
Neutropenia	1.6 [1.1-2.9]	0.002
Hypoalbuminemia	3.1 [1.6-4.9]	0.001
Hypovitaminosis D	1.9 [1.3-4.5]	0.001
High blood sugar	1.5 [1.1-5.3]	0.02
Methotrexate	0.6 [0.3-2.1]	0.60
Sulphasalazine	0.5 [0.4-2.3]	0.47
Hydroxychloroquine	0.3 [0.2-1.7]	0.41
Prednisolone <10 mg/ day	0.95 [0.7-3.5]	0.09
Prednisolone > 10 mg/day	2.1 [1.2-3.70]	0.01
Irregular Vaccination	1.3 [0.89-3.3]	0.07
Irregular follow up	0.7 [0.4-1.6]	0.11
Inadequate Counselling on Steroid	1.7 [1.1-3.9]	0.03

## Discussion

Infection is common among patients with rheumatological diseases who are on DMARD or biologicals. So it is of paramount importance to find out the pattern and factors associated with infection among those patients. This is the pioneering work as far as we know that has been done in Bangladesh that describes the pattern and predictors of infections in patients with different rheumatological diseases. We identified several predictors that can be associated with infection. Prior to a discussion of infection risk, it is important to remember that patients having rheumatological diseases are at risk of developing an infection in comparison to general people, independent of immunomodulatory drugs. Riley and George [5] describe herpes zoster, mycobacterial tuberculosis, and fungal infection as the most common infections observed among patients with rheumatoid arthritis on disease-modifying agents or biological agents or steroids. Dorgham et al. [6] also observed a similar pattern of infection that was mostly bacterially followed by fungal and viral infection among systemic lupus erythematosus (SLE) patients who were on DMARD or steroids. According to Germano et al. [7] most commonly observed infections were bacterial, followed by viral and fungal in both rheumatoid arthritis and spondyloarthropathy. Among the bacterial infection E. coli and Mycobacterium tuberculosis whereas herpes simplex and zoster were the most prevalent infections. We observed a similar pattern of infection until 2020. Thereafter, the viral infection that was COVID-19, was more prevalent among them. It has been a major concern since the beginning of the COVID-19 pandemic about the risk of SARS-CoV-2 infection and its complications in patients with systemic autoimmune diseases. The relationship between autoimmune disease and COVID-19 infection is quite complex and can be interpreted in different ways [8]. A key British study reported health data collected between February and 25 April 2020 on 17.5 million Britons with 5,683 hospital deaths due to COVID-19. Among the identified risk factors for mortality were autoimmune rheumatic disease [9]. Patients with autoimmune rheumatic diseases are at increased risk of COVID-19 incidence compared to the general population, immunosuppressive treatment, such as corticosteroids and other immune-modulators increase this risk. Most of the existing studies have not found associations between conventional synthetic disease-modifying antirheumatic drugs (csDMARD) or biologics and the risk of severe COVID-19 [10,11]. Some studies have even suggested a reduced risk of hospitalization in patients receiving tumour necrosis factor inhibitors (TNFi), although residual confounding selection bias could affect these findings [12]. In our opinion, as COVID-19 has high transmissibility, it invariably infects both healthy people and patients having rheumatic diseases. However, the severity of COVID-19 was observed among patients who were on prednisolone of more than 10mg per day for at least three months in our study. Still, the dosages of corticosteroids and their association with increased risk of infection are unknown. A case-control study by Dixon et al. modeled recent and prior glucocorticoid (GC) exposure and found a significant risk of infection with long-term doses ≤5mg/ day). Several observational cohort studies have demonstrated similar associations [13,14]. Among the conventional synthetic DMARD (methotrexate, sulfasalazine, leflunomide, and hydroxychloroquine) hydroxychloroquine has the best safety profile related to infection whereas meta-analyses of observational studies and randomized trials related to methotrexate have shown conflicting results; some have found no elevated risk of infection, serious infection or OI, but others have demonstrated a modest increase in risk [15,16]. We also observed a similar pattern of safety profile among csDMARD. Among the immunomodulators, cyclophosphamide and mycophenolate, and from the biological group tofacitinib were associated with more infection than other agents in our study. It is interesting that in our study serious infections were not observed in anti-TNF (adalimumab, etanercept) alone group. However, patients who received anti-TNF along with steroids suffered more serious infections. Riley and George [5], Germano et al. [7], and Doran et al. [17] described similar patterns of infections among patients on DMARD and biological agents.

Lin et al. [18] analyzed 24 studies published prior to May 2015 whose focus was the relationship between serum 25-OHD3 and clinical/laboratory indices of rheumatoid arthritis (RA) disease activity. Overall, they reported an inverse relationship between serum 25-OHD3 and RA disease activity. Low vitamin D might be the consequence of chronic inflammation, leading to a dysregulated immune system making a person more prone to infection [19]. Vitamin D levels were low in most of our patients who suffered infections. Although most of them were female, adequate sun exposure was ensured. Most of our patients had hypoalbuminemia, and leukopenia and were underweight. Innate and adaptive immunity depends on albumin. Therefore, hypoalbuminemia is associated with the acquisition and severity of infectious diseases. There is a causal link between hypoalbuminemia and an increased risk of infections. Serum albumin levels and leucopenia have prognostic value for complications in viral, bacterial, and fungal infections, and for infectious complications of non-infective chronic conditions [20,21]. Surprisingly, patients who were counseled about the side effects of steroids suffered less serious infections. So, counseling about the side effects of steroids must be ensured while prescribing steroids.

Although the present study was conducted from the recorded data of a rheumatology clinic, however, all patients with documented infections were enrolled. Logistic regression was used to find out the predictors. Present study had some limitations. Those were retrospective study design, small sample size, and single-centered study. As the number of infected patients number was small that’s why regression was not possible to conduct among cyclophosphamide, mycophenolate, and tofacitinib groups. So it demands a multicenter large-scale study to find a causal association of infections with the predictors.

## Conclusions

COVID-19 pneumonia, urinary tract and local fungal infections, tuberculosis, herpes zoster, and community-acquired pneumonia were commonly occurring infections among patients receiving different immunosuppressive medications. Factors like poor nutritional status, presence of anemia, leucopenia, hypoalbuminemia, hyperglycemia, and hypovitaminosis D had a significant association with infection. Moreover, inadequate counseling of steroid side effects and history of daily intake of prednisolone (>10mg/day) were also significant factors associated with infection.

## References

[1] Bulpitt KJ (1999). Biologic therapies in rheumatoid arthritis. Curr Rheumatol Rep.

[2] Quartuccio L, Zabotti A, Del Zotto S, Zanier L, De Vita S, Valent F (2019). Risk of serious infection among patients receiving biologics for chronic inflammatory diseases: usefulness of administrative data. J Adv Res.

[3] Singh JA, Cameron C, Noorbaloochi S (2015). Risk of serious infection in biological treatment of patients with rheumatoid arthritis: a systematic review and meta-analysis. Lancet.

[4] Kourbeti IS, Ziakas PD, Mylonakis E (2014). Biologic therapies in rheumatoid arthritis and the risk of opportunistic infections: a meta-analysis. Clin Infect Dis.

[5] Riley TR, George MD (2021). Risk for infections with glucocorticoids and DMARDs in patients with rheumatoid arthritis. RMD Open.

[6] Dorgham DA, Anwar S, Khaled AS (2021). Infection in systemic lupus erythematosus patients. Egypt Rheumatol.

[7] Germano V, Cattaruzza MS, Osborn J, Tarantino A, Di Rosa R, Salemi S, D'Amelio R (2014). Infection risk in rheumatoid arthritis and spondyloarthropathy patients under treatment with DMARDs, corticosteroids and TNF-α antagonists. J Transl Med.

[8] Haberman RH, Castillo R, Chen A (2020). COVID-19 in patients with inflammatory arthritis: a prospective study on the effects of comorbidities and disease-modifying antirheumatic drugs on clinical outcomes. Arthritis Rheumatol.

[9] Williamson EJ, Walker AJ, Bhaskaran K (2020). Factors associated with COVID-19-related death using OpenSAFELY. Nature.

[10] Favalli EG, Agape E, Caporali R (2020). Incidence and clinical course of COVID-19 in patients with connective tissue diseases: a descriptive observational analysis. J Rheumatol.

[11] Pablos JL, Abasolo L, Alvaro-Gracia JM (2020). Prevalence of hospital PCR-confirmed COVID-19 cases in patients with chronic inflammatory and autoimmune rheumatic diseases. Ann Rheum Dis.

[12] Gianfrancesco M, Hyrich KL, Al-Adely S (2020). Characteristics associated with hospitalisation for COVID-19 in people with rheumatic disease: data from the COVID-19 Global Rheumatology Alliance physician-reported registry. Ann Rheum Dis.

[13] Dixon WG, Abrahamowicz M, Beauchamp ME, Ray DW, Bernatsky S, Suissa S, Sylvestre MP (2012). Immediate and delayed impact of oral glucocorticoid therapy on risk of serious infection in older patients with rheumatoid arthritis: a nested case-control analysis. Ann Rheum Dis.

[14] Wilson JC, Sarsour K, Gale S, Pethö-Schramm A, Jick SS, Meier CR (2019). Incidence and risk of glucocorticoid-associated adverse effects in patients with rheumatoid arthritis. Arthritis Care Res (Hoboken).

[15] Suarez-Almazor ME, Belseck E, Shea B, Wells G, Tugwell P (2000). Methotrexate for rheumatoid arthritis. Cochrane Database Syst Rev.

[16] Ibrahim A, Ahmed M, Conway R, Carey JJ (2018). Risk of infection with methotrexate therapy in inflammatory diseases: a systematic review and meta-analysis. J Clin Med.

[17] Doran MF, Crowson CS, Pond GR, O'Fallon WM, Gabriel SE (2002). Predictors of infection in rheumatoid arthritis. Arthritis Rheum.

[18] Lin J, Liu J, Davies ML, Chen W (2016). Serum vitamin D level and rheumatoid arthritis disease activity: review and meta-analysis. PLoS One.

[19] Mangin M, Sinha R, Fincher K (2014). Inflammation and vitamin D: the infection connection. Inflamm Res.

[20] Wiedermann CJ (2021). Hypoalbuminemia as surrogate and culprit of infections. Int J Mol Sci.

[21] Klein A, Molad Y (2021). Hematological manifestations among patients with rheumatic diseases. Acta Haematol.

